# Intrasaccular therapy in wide-neck intracranial aneurysms: a narrative review

**DOI:** 10.3389/fneur.2025.1552848

**Published:** 2025-07-01

**Authors:** Kaiquan Zhuo, Hongxia Wu, Zhongji Gou, Changwei Zhang

**Affiliations:** ^1^Department of Neurology, The People’s Hospital of Pengzhou, Pengzhou, China; ^2^Department of Respiratory and Critical Care Medicine, West China Hospital, Sichuan University, Chengdu, China; ^3^Department of Neurosurgery, West China Hospital, Sichuan University, Chengdu, China

**Keywords:** intracranial aneurysm, wide-neck aneurysms, endovascular procedure, intrasaccular device, interventional therapy

## Abstract

The treatment of wide-neck intracranial aneurysms (WNIAs) is a challenge in interventional therapy. Intrasaccular therapy, including intrasaccular flow disruption or formation of a neck bridge within the saccular, has been extensively used recently. The Medina® Embolisation Device, Luna™ Aneurysm Embolisation System, Artisse™ Aneurysm Embolisation System, Contour Neurovascular System™, Neqstent™ Coil Assisted Flow Diverter, Woven EndoBridge (WEB) device, Nautilus™ Intrasaccular System, and Trenza Embolization Device™ have obtained Conformité Européene Mark certification. However, only WEB devices have been approved by the US Food and Drug Administration. The SEAL™ Endovascular Embolization System is approved for sale only in New Zealand. Angiographic results post-procedure and in the follow-up are the main indices used to measure the efficacy of intrasaccular therapy. Complications include ischaemia, thromboembolism, and related haemorrhage. This review summarizes and discusses the efficacy and safety of intrasaccular devices in treating WNIAs. Despite this, complete and satisfactory occlusions are accomplished in most cases of intrasaccular therapy for WNIAs, and the associated complications are typically regarded as acceptable. However, it is important to note that the occlusion rate with intrasaccular therapy is generally lower than that achieved through surgical clipping. Long-term follow-up of intrasaccular therapy and recurrence and retreatment of WNIAs is limited.

## 1 Introduction

Intrasaccular treatment of intracranial aneurysms (IAs) involves the insertion of a flow disruptor or forming a neck bridge to offer favourable stability compared to coiling alone. Balloon- or stent-assisted coiling and flow diversion are the primary endovascular treatments for wide-neck intracranial aneurysms (WNIAs). However, complex stent configurations, the risk of covering bifurcation branches or obstructing parent vessels, and antiplatelet therapy have led to the innovation of new intrasaccular therapies for IAs. The Woven EndoBridge (WEB) device (Sequent Medical, Aliso Viejo, CA, USA, now Terumo Neuro, Aliso Viejo, CA, USA), an intrasaccular flow disruption device, was the first intrasaccular technique used to treat WNIAs and was first utilised in 2010 (1). Intrasaccular therapy does not involve metal stents within the parent vessel or antiplatelet therapy, thus reducing the risk of ischaemia, thromboembolism, and haemorrhage.

Scholars discussed the definition of a WNIA, and the most prevalent definition is a neck diameter of ≥4 mm or a dome-to-neck ratio of <2 (2). Angiographic results are assessed using the Raymond–Roy or modified Raymond–Roy occlusion classification, an angiographic classification tool for grading the occlusion of aneurysms treated with embolisation (3). Classes I and II are complete obliteration and residual neck, respectively. Complete occlusion refers to class I, and adequate occlusion refers to classes I and II.

To date, eight devices have received Conformité Européene (CE) Mark certification: Medina® Embolisation Device (Covidien, Irvine, CA, USA), Luna™ Aneurysm Embolisation System (Medtronic, Minneapolis, MN, USA), Artisse™ Aneurysm Embolisation System (Medtronic, Minneapolis, MN, USA), Neqstent™ Coil Assisted Flow Diverter (Cerus Endovascular, Fremont, CA, USA, now Stryker neurovascular, Kalamazoo, MI, USA), Contour Neurovascular System™ (Cerus Endovascular, now Stryker neurovascular, Kalamazoo, MI, USA), WEB device, Nautilus™ Intrasaccular System (EndoStream, Or Akiva, Israel), and Trenza embolization device™ (Stryker neurovascular, Kalamazoo, MI, USA) (4, 5). However, only the WEB device has been approved by the US Food and Drug Administration (FDA) (6). Additionally, the The Saccular Endovascular Aneurysm Lattice (SEAL™) Endovascular Embolization System (Galaxy Therapeutics Inc., Milpitas, CA, USA) is approved for sale only in New Zealand (7). This review summarises and discusses the efficacy and safety of intrasaccular therapy for WNIAs.

## 2 Methods

We searched Web of Science, Medline, Cochrane, and Embase using the following terms: intracranial aneurysm, wide-neck, endovascular procedure, and intrasaccular device from database inception to May 1, 2025. The following criteria were used to determine the suitability of studies for inclusion: (1) Randomized controlled trials (RCTs), as well as cohort studies, case reports, and case series. (2) Studies that reported data on patients with both ruptured and unruptured WNIAs treated with intrasaccular therapy, including information on angiographic classification. (3) The definition for WNIAs included a neck diameter of≥4 mm or a dome-to-neck ratio of <2. Patients who were admitted for other medical issues, those who had undergone surgical craniotomy, individuals with severe dysfunction of other organs or systems, and patients with malignant tumors were excluded from the study. Additionally, studies involving pregnant subjects were not considered. We reviewed all retrieved literature’s abstracts to identify suitable trials. We also retrieved the references from all the literature to minimise error and bias. This review is based on previous studies and contains no new studies with human participants or animals performed by any authors.

## 3 Results

### 3.1 Medina embolisation device

**Figure 1:**
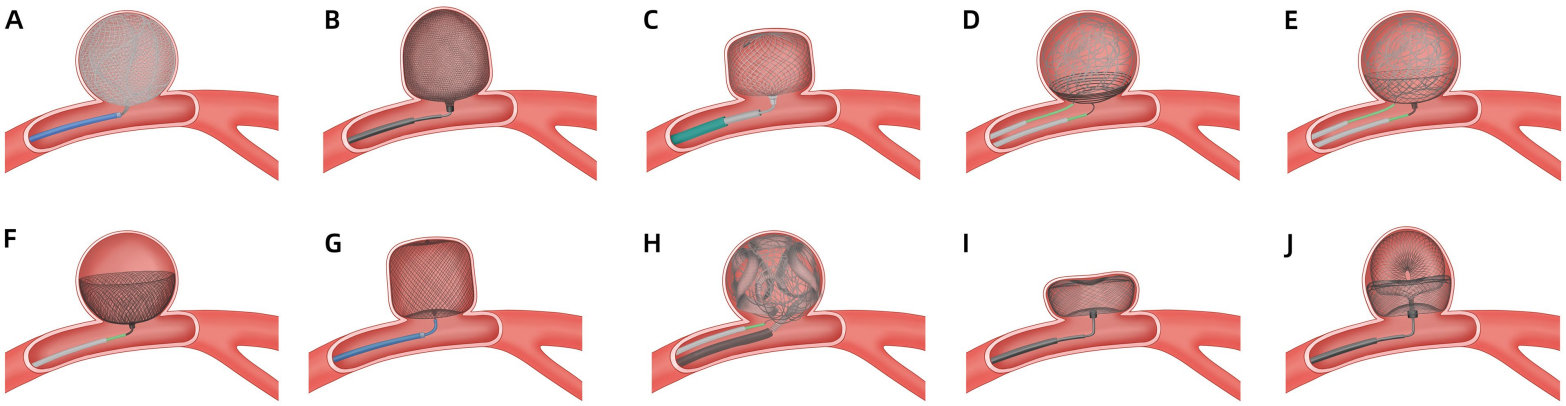
Images of the intrasaccular devices embolization of a wide-neck intracranial aneurysm.

The Medina is a mechanically detached, braided, self-expanding device comprising a radiopaque three-dimensional set core wire and a wing-like shape memory alloy filament that forms a low-porosity metal mesh (Figure 1A) (8). The Medina is applied via a 0.021-inch diameter microcatheter and can be re-sheathed and redeployed, as with standard coils. Aguilar Perez et al. (9) successfully applied a Medina device in 14 patients with unruptured aneurysms. Immediate and 6–12 months angiography results showed complete and adequate occlusion rates of 6.7, 42.9, 36.4, and 54.5%, respectively. No complications occurred. However, adjunctive devices are required in wide-neck cases. Sourour et al. (10) evaluated Medina’s safety and 6–9-month efficacy in 12 patients with 13 WNIAs. In 15% of cases, Medina was used alone. However, 85 and 31% of cases used additional coils or adjunctive balloons, respectively. Postprocedure and 6-month complete occlusion rates were 61.5 and 83%, respectively. One patient with no clinical consequences of thromboembolic complications was observed. Bhogal et al. (11) reported 14 aneurysms in 13 patients treated with Medina. The aneurysm neck width ranged from 1.9 to 6.9 mm. A complete occlusion rate was observed in 15.4% of the cases, and an adequate occlusion rate in 23.1%. Five months after angiography, the complete and adequate occlusion rates were 38.5 and 76.9%, respectively. Seventy-five percent of cases treated with two or more Medina had complete occlusion at 6 months, and three patients experienced temporary complications. A study reported angiographic outcomes in 19 patients with 20 WNIAs treated with Medina (12). The post-procedure, 6-month, and long-term follow-up complete occlusion rates were 61, 75, and 80%, respectively. Angiographic recanalisation was documented in 11% of the cases at mid-term follow-up. Three cases (15%) of thromboembolic complications were observed; however, only one was responsible for clinical sequelae. No recanalisation occurred during the long-term follow-up. Compared with the other devices, the Medina had relatively poor immediate, mid-term, and long-term efficacy, and outcomes highly depended upon the number of devices or adjunctive devices. Due to the high cost of using multiple devices and the difficulty of reoperation, the device was not used clinically.

### 3.2 Luna aneurysm embolisation system

The Luna is a pre-shaped, ovoid, self-expanding, mechanically detachable endovascular device made from a double layer of wire mesh designed to provide coverage across the aneurysm neck and isolate the saccular artery from the parent artery (Figure 1B). Its size (4.5–8.5 mm) is based on the aneurysm width, shape, and dome-to-neck ratio. The device can be inserted via a microcatheter with a 0.027-inch lumen. Piotin et al. (13) evaluated the efficacy and safety in 63 bifurcation and sidewall IAs, of which 95.2% were unruptured. The neck sizes were 1.9–8.7 mm, and the immediate adequate occlusion rate was 18%. However, adequate occlusion rates were achieved in 78.0 and 79.2% of patients at 12 and 36 months, respectively. Two major strokes (3.2%), one minor stroke (1.6%), and three incidents of haemorrhage in two subjects (3.2%) were observed. No morbidities were observed after 12 months of treatment. Morbidity was 1.8% at 36 months, and one case of mortality (1.6%) was observed. Kwon et al. (14) demonstrated that the Luna may not be suitable for fusiform, wide-neck, or complex aneurysms, as few WNIAs were included in the study reported by Piotin (13). Due to the appearance of the Artisse, Luna is no longer in clinical use.

### 3.3 Artisse aneurysm embolisation system

The Artisse is a second-generation Luna device. It is a flex-to-fit intrasaccular device that conforms to the aneurysm’s shape (Figure 1C) (15). This device is intended for the endovascular embolization of saccular intracranial aneurysms. Its width and height diameter range from 4.5 to 8 mm and 3.0 to 5.0 mm, respectively. The device has 20 sizes. The device can be inserted via a microcatheter with a 0.021-inch lumen. The device detaches through an electrolytic, handheld detachment system. Piotin et al. (16) reported the outcomes of patients with unruptured bifurcation aneurysms treated with Artisse. The mean aneurysm neck size was 4.0 ± 0.8 mm (range 3.2–5.8 mm), and the dome-to-neck ratio was 1.82:1. Adequate occlusion was achieved in 66.7 and 57.1% of patients at 6 and 36 months of follow-up, respectively. Of the 9 participants, 22.2% experienced major strokes, including one procedure-related parent vessel occlusion and one haemorrhagic stroke. Due to the small sample size of the study, further research is necessary to confirm the results. Besides, more research is needed to guide the selection of appropriate device sizes, as a great variety of sizes is available.

### 3.4 Nautilus intrasaccular system

The Nautilus is a novel device designed for neck bridging, a technique used to enhance coil stability and reduce the risk of coil protrusion in WNIAs (Figure 1D) (5). The device is a nitinol-based detachable disk-like implant with flexible layers that can be delivered via a 0.017-inch microcatheter. The device is fully retrievable and can be repositioned at any time. The coils are delivered to the aneurysm using another microcatheter. The suitable device size is at least 0.5 mm larger than the width of the neck. Several case reports have described the successful application of the Nautilus in IAs (17–19). Recently, Sirakov et al. (20) reported the outcomes in 41 patients with WNIAs treated with the Nautilus. Immediate and follow-up complete occlusion rates were 73.1 and 94.5%, respectively. No procedure-related deaths or morbidities were reported. For immediate aneurysm dome protection, the adjunctive coiling technique enhances its application in treating acutely ruptured aneurysms. The results of the Nautilus are promising, but further long-term studies are required. These studies have limitations such as small sample size, being a single-center series, retrospective analysis without a comparator group, and a short follow-up period.

### 3.5 Neqstent coil assisted flow diverter

The Neqstent is a neck-bridging, electrolytically detachable, self-expanding device used in conjunction with coiling or other embolisation products to facilitate the occlusion of WNIAs (Figure 1E) (21). It treats a wide range of unruptured aneurysm morphologies including wide-necked bifurcation and bifurcation aneurysms. The concave-shaped device has 64 wires with a diameter of 0.0015 inches and can pass through a 0.017-inch microcatheter for coiling. The Neqstent is a derivative of the Contour that may be applied to ruptured IAs. Four sizes (7, 9, 11, and 14 mm) are suitable for 3–10 mm neck-width IAs. The device is delivered via a 0.021–0.027-inch microcatheter. The Neqstent was implanted in 36/38 unruptured WNIAs in a prospective multicentre study (22). The median neck length of the aneurysm was 5.2 mm (range 2.1–11.4 mm). The immediate adequate occlusion rate was 25%, which increased to 77.8% at 6 months. Complete occlusion was achieved in 80.6% of patients at the last angiography. Related complications were reported in 10.5% of the patients, including one haemorrhagic event and three thromboembolic events. The primary limitations include the sample size, lack of randomization, and the inclusion of patients with previous aneurysm treatment. The Neqstent remains within the neck of the aneurysm, allowing the coil to be fixed without needing a stent. The additional flow-directing characteristics may enhance the new endothelialization. Although 0.017-inch microcatheters can pass through the reticular structure on the side of the Neqstent, the best technique for locating coil microcatheters into aneurysms is jailing.

### 3.6 Contour neurovascular system

The Contour is easily re-sheathable and re-deployable, and is a neck-bridging device (Figure 1F) (23, 24). The device expands to a cup-like shape to fill the proximal half of the IA. The Contour, which has a dual-layer conformation with 144 wires, has both the effect of flow diversion and disruption. Size selection (5, 7, 9, 11, and 14 mm) is based on the IA width and neck width, which range from 2 mm to 10.5 mm and 2 mm to 10 mm, respectively. The Contour can be deployed via a 0.021-inch or 0.027-inch microcatheter. A review reported three patients with three unruptured aneurysms treated with the Contour (25). The neck widths of 2 IAs were >4 mm. One patient had incomplete occlusion post-procedure, and 6 months later. One patient had complete occlusion at 3 and 15 months. Akhunbay-Fudge et al. (26) reported outcomes of 11 patients with WNIAs treated with the Contour. The complete and adequate occlusion rates at 1 year were 55.56 and 100%, respectively. Two patients had thromboembolic events but without permanent neurological disability or death. Liebig et al. (27) reported the safety and efficacy outcomes of 32 patients with unruptured intracranial bifurcation aneurysms who received treatment with the Contour. The median aneurysm neck width was 4.3 ± 1.4 mm (range 2.4–7.4 mm) and the dome-to-neck ratio was 1.4 ± 0.4. Complete occlusion was observed in 44 and 69% of the patients at 6 and 12 months, respectively. The adequate occlusion rate was 84% at the final follow-up. Ghozy et al. (28) reported that 131 patients with WNIAs were treated using a Contour. The occlusion rate was 84.21%, and the thromboembolic event rate was 8.53%. Mostafa et al. (29) reported that complete and adequate occlusion rates were 20 and 100%, respectively, at 6 months in 5 patients with wide-neck basilar tip aneurysms treated with a Contour device. Diana et al. (30) studied the safety and feasibility of Contour and Neqstent in 15 cases of IAs. The neck width in 6 cases was >4 mm. In one case, deployment failed because of the parent artery’s stenosis; in another case, balloon-assisted coiling was selected to protect against coil protrusion. One ischaemic event (6.7%) was observed, and no haemorrhagic complications, mortality, or mobility were reported. Overall, the Contour is a safe and effective treatment option for WNIAs. Sizing is simpler than the WEB, which offers only five sizes. The slightly oversized design guarantees secure placement without any unintended movement after deployment.

### 3.7 Woven EndoBridge device

The WEB device, introduced in 2010, is the first and only FDA-approved device for intrasaccular flow disruption in ruptured and unruptured aneurysms (Figure 1G) (31). The WEB device is indicated for use at the internal carotid artery terminus, middle cerebral artery bifurcation, anterior communicating artery complex, or basilar artery apex for endovascular treatment with saccular, wide-neck bifurcation ruptured and unruptured IAs with a dome diameter of 3–10 mm and either neck size ≥4 mm or a dome-to-neck ratio 1–2. During treatment, the physician selects the appropriate device size based on the size, shape and location of the IA to be occluded. The WEB is an electrothermally detached braided nitinol cage-like device, which includes three types: dual-layer (WEB-DL), single-layer (WEB-SL), and single-layer spherical lower-profile (WEB-SLS). However, WEB-DL is no longer commercially available. The evolution from WEB-DL to WEB-SL balances porosity, radial force, and thrombogenicity (32). The device can be delivered using 0.017-, 0.027-, or 0.033-inch microcatheters. The WEB device causes rapid thrombosis and occlusion of the aneurysm, leading to immediate alteration of haemodynamics. The WEB device has become widely used in wide-neck bifurcation aneurysms following the WEB-Intrasaccular Therapy (WEB-IT) prospective study (33). A 10-year study on WEB application post-procedure and long-term angiographic efficacy reported no variations in complications or mortality (34). Fujii et al. (35) reported the WEB use in 29 patients with WNIAs. The mean neck width was 3.7 ± 0.6 mm, and the adequate occlusion rate was 75.9% after 6 months. WEB shape modification was a contributing factor for adequate occlusion, as identified by multivariate analysis. One patient had a renal artery injury, and another had a minor cerebral infarction. Mantilla et al. (36) conducted a systematic review of 22 articles, including 1705 patients with 1,224 aneurysms, and discussed the application of WEB devices in IAs, predominantly WNIAs. The immediate and follow-up adequate occlusion rates were 33.3 and 49.7%, respectively. A total of 6.5 and 3.1% of patients had thromboembolic complications and other complications, respectively. The mortality rate was approximately 1%. However, Morioka et al. (37) reported persistent contrast-filling in the WEB in 50% (10/20) of IAs after 3 months. Persistent contrast filling was associated with postoperative dual antiplatelet therapy for at least a month, a wide neck (median 4.5 mm), and a lower deviation of the aneurysm axis from the inlet flow line.

The current data suggest that the WEB device is most suitable for treating typical wide-necked and bifurcated aneurysms due to its compact structure and variable size. However, it may not be effective for aneurysms with a shallow depth, very irregular shapes, or those requiring secondary treatment. Additionally, the rigidity of the delivery system and the device itself may pose challenges in accurately placing it. Selecting the appropriate size is a significant issue. Compared with Contour, which only relies on the maximum width diameter for selection, correctly choosing the WEB size is more challenging. The WEB is more likely to be too small, explaining some recurrence and retreatment cases.

### 3.8 Trenza embolization device

The Trenza embolization device is a frame coil implant that creates a stable *ω*-shaped basket filled with coils with flow-disruption properties to treat challenging mid-to-large-sized broad-neck bifurcation or sidewall aneurysms (Figure 1H) (38). It is available in 6- to 12-mm sizes, each with a fixed length (eg, 6–11 mm, 7–13 mm, 8–15 mm, and so forth). The device is attached to a flexible delivery wire, and the detachment of the device is electrothermal. An observational single-center retrospective study reported the experience of 12 patients (10 unruptured and 2 ruptured) with WNIAs treated by the Trenza device (38). The median dome-to-neck ratio was 1.8 (interquartile range, 1.6–1.9). Complete and adequate occlusion was 67 and 83% at the 6.5-months follow-up. There were 3 (25%) major ischemic complications, leading to 2 (17%) permanent and 1 (8%) transient neurologic deficit. Two (17%) patients were retreated. One (8%) treated aneurysm ruptured 1.6 months after follow-up and resulted in death. A retrospective multicenter analysis reported the results of a 6-month follow-up involving 25 aneurysms (3 ruptured) in 25 patients who received the Trenza device (39). Twenty-one cases met the criteria for WNIAs. The rates of complete and adequate occlusion were 42.1 and 89.5% after 6 months. Additionally, 10.5% of cases required retreatment. There was one reported symptomatic thrombotic event, and two cases received stent assistance. The limitations of these two studies, due to the retrospective design and a relatively small number of included patients. More studies are needed.

### 3.9 The SEAL endovascular embolization system

The SEAL Embolization System is a novel intrasaccular aneurysm low-disruptor (7). The SEAL is a self-expanding, dual-layer, nitinol, and core platinum wire mesh braided implant. The SEAL has two configurations: one configuration has only the base portion (Figure 1I) (SEAL Base), and the second configuration includes an ovoid upper loop with a base bridging component (Figure 1J) (SEAL Arc). Pabon et al. (7) present the first case report of SEAL demonstrated in a patient with acute hemorrhage from a ruptured, complex, left middle cerebral artery trilobed shallow wide-necked bifurcation aneurysm. Immediate and a 1-year angiographic follow-up demonstrated complete occlusion with no safety concerns. The SEAL device is only approved for marketing and commercialization in New Zealand. This case report is part of the Pre-SEALIT ethics committee-approved early feasibility clinical study in Colombia. The SEAL device may be a promising novel technology that can potentially treat very shallow aneurysms with limited height and irregular, multilobulated aneurysms.

## 4 Discussion

The characteristics of intrasaccular devices are summarized in Table 1. The available research indicates that intrasaccular devices are suitable for typical WNIAs because of their variable size and compact nature. However, deploying intrasaccular devices within WNIAs with shallow depths, irregular shapes, and multilobular or pretreated aneurysms presents some challenges (40). The SEAL device may be a promising device to treat very shallow aneurysms (7). Trimboli et al. (41) studied the safety and efficacy of balloon-assisted WEB deployment in 33 IAs, of which 25 aneurysms (75.8%) were classified as WNIAs. At the mid-term follow-up, complete and adequate occlusion rates were 85.2 and 92%, respectively. One (3.0%) patient died of procedure-related complications. Goertz et al. (42) reported the outcomes of 178 IAs treated with WEB or stent-assisted WEB devices. Stent implantation was performed in 15 patients (8.4%). The thromboembolic complication rate was higher in the stent-associated WEB group (33.3% vs. 8.0%), whereas ischaemic stroke rates were comparable between the groups (0% vs. 1.8%). At the 6-month follow-up, the complete and adequate occlusion rates were 73.4 and 92.8% after WEB and 66.7 and 86.7% after stent-associated WEB, respectively.

**Table 1:** Characteristics of the intrasaccular devices.

Device	Company	Techinical characteristics	Device sizes (mm)	Indications	Aneurysm size (mm)	Microcatheter inner diameter (Inches)	Adjunctive therapy	Detachment	Availability
Medina	Covidien	Intrasaccular disruption	5–9	All saccular aneurysms	More than 5	0.021	None	Mechanically	Unavailable
Luna	Medtronic	Intrasaccular disruption	4.5–8.5	Saccular intracranial bifurcation and sidewall aneurysms	Height: 4.7–12.6, width: 3.0–8.5	0.021	None	Mechanically	Unavailable
Artisse	Medtronic	Intrasaccular disruption	Width:4.5–8.0Height: 3.0–5.0	Intracranial aneurysms	Height: 5.0–8.0	0.021	None	Electrolytically	Available
Nautilus	EndoStream Medical	Neck-bridging	4, 5, 6, 7	Wide-neck intracranialaneurysms	Neck<6.5	0.0165	Coiling	Electrolytically	Available
Neqstent	Stryker neurovascular	Neck-bridging, flow diversion	7,9,11, 14	Unruptured aneurysms including wide-necked bifurcation and bifurcation aneurysms	Neck: 3–10	0.021, 0.027	Coiling	Electrolytically	Available
Contour	Stryker neurovascular	Flow diversion, flow disruption	5, 7, 9, 11, 14	Intracranial aneurysms	Width: 2–10.5, neck: 2–10	0.021, 0.027	None	Electrolytically	Available
Woven EndoBridge	Terumo Neuro	Intrasaccular disruption	4–11	Unruptured and rupture bifurcation intracranial aneurysms	Dome:3–10 and either neck≥4 or <1 dome-to-neck <2 ratio	0.017, 0.021, 0.027, 0.033	None	Electrolytically	Available
Trenza	Stryker neurovascular	Intrasaccular disruption	6–12	Wide-neck bifurcation or sidewall aneurysms	Neck: 6–12 and either neck≥4 or <1 dome-to-neck <2 ratio	0.019	Coiling	Electrolytically	Available
SEAL	Galaxy therapeutics	Intrasaccular disruption	3–20	Shallow, irregular, multilobulated aneurysms; deep, elongated, andspherical shape aneurysms	Width: 2.5–16.5	0.017, 0.021, 0.027, 0.033	None	Electrolytically	Available

NM, not mentioned.

Appropriate sizing selection is a major issue in all intrasaccular therapies. Studies associated with the Medina device have demonstrated that using more than one device is associated with an improved occlusion rate. However, the method is expensive. Compared with the Contour, which only requires the largest diameter, the sizing of WEB devices is difficult. Therefore, the WEB is more prone to undersizing, which may influence the recurrence and retreatment rates of IAs. Goertz et al. (43) performed a retrospective multicentre study of 247 patients with 251 IAs treated using WEB device oversizing. Of 237 aneurysms, 94.4% were WNIAs, with median neck width and dome-to-neck ratios of 4.6 ± 1.4 mm (range 1.2–10.3 mm) and 1.4 ± 0.4 mm (0.6–3.4 mm). Ruptured aneurysms were present in 25.5% of cases (n = 64). The WEB device was successfully applied in 98.8% of patients, achieving a mean WEB/dome ratio of 1.2 ± 0.1. The midterm complete and adequate occlusion rates were 66.3 and 88.4%, respectively. The thromboembolic complication rate was 7.2%. Complications were reported in eight procedures (3.2%), the morbidity rate was 0.8%, and no mortalities were observed in this study. Oversizing the WEB device may yield promising complete and adequate mid-term occlusion rates. One study (n = 120) demonstrated that an increased width-to-height ratio and a lower aneurysm height/neck diameter ratio were significant risk factors for complications in WEB therapy (44). Due to the vary sizes, it’s difficult to determine the correct size without any prior experience. In an animal model, Sim&Cure software was used to run various simulations and assist to select the right size of Artisse (45). Jadhav et al. (46) already introduced a machine learning model to help predict the outcome of treatment with a WEB device for WNIAs. Artificial intelligence could play a crucial role in helping us select more suitable device types and sizes in the future.

Previous studies have reported that surgical clipping has a complete occlusion rate of up to 96% (47). Comparatively, the rate of residual aneurysm or recurrence ranges from 60 to 95% with endovascular treatment. Recurrence and retreatment rates are higher in patients with larger or wide-neck aneurysms. Initial partial aneurysm thrombosis, aneurysm size, and simultaneous treatment with WEB and a coil were associated with higher recurrence and retreatment rates (48). Cagnazzo et al. (49) reported that aneurysm rupture, undersized WEB, WEB shape change, neck diameter, and angle between the parent artery and aneurysm dome were associated with a lower adequate occlusion rate. However, the results were insignificant. A recent study on the retreatment of residual and recurrent aneurysms treated with WEB suggested that a neck remnant might not be adequate, and cases must be followed up closely (50). Although thrombotic events also exist, the rates of residual and recurrent aneurysms are significantly higher than those of thrombotic events. The surface coating of the device that encourages thrombosis or endothelialization may be beneficial in improving the rates of complete or adequate occlusion.

The different intrasaccular devices used in WNIAs demonstrate varying degrees of efficacy and safety. Hecker et al. (51) compared a Contour device (34 patients with 40 aneurysms) with a WEB device (30 patients with 30 aneurysms) for the treatment of WNIAs. In the Contour cohort, the complete and adequate occlusion rates at the last follow-up were 75 and 90%, respectively. One patient (2.5%) required retreatment, and one patient experienced a symptomatic thromboembolic event. Three adjunctive stents (10%) were used for branch occlusion. In the WEB cohort, the complete and adequate occlusion rates were 63.3 and 90%, respectively, with a retreatment rate of 20%. Four (13.3%) patients required an additional stent because of device protrusion into a branch. Two asymptomatic thromboembolic events (6.7%) and one major ischaemic event (3.3%) were reported. The Contour cohort outcomes were promising, although a more extended follow-up period is necessary. Several factors can influence the occlusion rate of WNIAs when an embolization device is implanted. These factors include the availability, morphology, structure, and material properties of the devices, as well as the location and shape of the WNIAs. Consequently, different studies may report varying occlusion rates. However, further RCTs are needed to validate these findings.

The outcomes of microsurgical clipping and endovascular techniques in WNIAs vary. A review found that lipping is linked to a higher rate of occlusion and lower rates of residual and recurrent aneurysms. In contrast, coiling results in lower morbidity and mortality, along with a more favorable postoperative outcome (47). In comparing the efficacy and safety of WEB (63 patients) and clipping (103 patients), microsurgical clipping was associated with a higher complete occlusion rate. In contrast, the WEB technique demonstrated a lower complication rate (52). This study included unruptured anterior circulation aneurysms. Complications occurred in 13% of patients in the clipping group and 6% in the WEB group. A retrospective study that included 84 patients with 89 unruptured WNIAs compared the efficacy and safety of WEB with those of microsurgical clipping (53). Of the WNIAs, 99% were completely occluded with clipping compared with a WEB occlusion rate of 53% at 1 year follow-up. The morbidity rates in the microsurgical clipping and WEB groups were 1 and 3%, respectively. This retrospective study involved patients with unruptured WNIAs, as per the criteria for WEB. Fusiform, multilobulated, and dissecting aneurysms were excluded. The inclusion criteria for these studies varied, making it impossible to demonstrate the superiority of WEB intrasaccular therapy over microsurgical clipping. Therapeutic decisions should be made based on individual preferences. In addition, compared with traditional coil or coil-assisted therapy, the higher costs of intrasaccular treatment might limit its use as the first choice of treatment for WNIAs.

## 5 Conclusion

With innovations in intrasaccular devices, the safety and occlusion rate of intrasaccular treatments for WNIAs are promising. However, most current research focuses primarily on unruptured WNIAs; long-term follow-up and outcome data are lacking. Future studies on ruptured WNIAs, the comparison of intrasaccular therapy with microsurgical clipping, and long-term outcomes are needed.
